# A-agents, misleadingly known as “Novichoks”: a narrative review

**DOI:** 10.1007/s00204-023-03571-8

**Published:** 2023-08-24

**Authors:** Jakub Opravil, Jaroslav Pejchal, Vladimir Finger, Jan Korabecny, Tomas Rozsypal, Martina Hrabinova, Lubica Muckova, Vendula Hepnarova, Jan Konecny, Ondrej Soukup, Daniel Jun

**Affiliations:** 1 Faculty of Military Health Sciences, University of Defence, Trebesska 1575, 500 01 Hradec Kralove, Czech Republic; 2 Faculty of Pharmacy in Hradec Kralove, Charles University, Akademika Heyrovskeho 1203, 500 05 Hradec Kralove, Czech Republic; 3 Biomedical Research Center, University Hospital Hradec Kralove, Sokolska 581, 500 05 Hradec Kralove, Czech Republic; 4 Nuclear, Biological and Chemical Defence Institute, University of Defence, Vita Nejedleho 1, 682 03 Vyskov, Czech Republic

**Keywords:** A-series agents, Physicochemical properties, Environmental stability, Toxicity, Analysis, Therapy

## Abstract

“Novichok” refers to a new group of nerve agents called the A-series agents. Their existence came to light in 2018 after incidents in the UK and again in 2020 in Russia. They are unique organophosphorus-based compounds developed during the Cold War in a program called Foliant in the USSR. This review is based on original chemical entities from Mirzayanov's memoirs published in 2008. Due to classified research, a considerable debate arose about their structures, and hence, various structural moieties were speculated. For this reason, the scientific literature is highly incomplete and, in some cases, contradictory. This review critically assesses the information published to date on this class of compounds. The scope of this work is to summarize all the available and relevant information, including the physicochemical properties, chemical synthesis, mechanism of action, toxicity, pharmacokinetics, and medical countermeasures used to date. The environmental stability of A-series agents, the lack of environmentally safe decontamination, their high toxicity, and the scarcity of information on post-contamination treatment pose a challenge for managing possible incidents.

## Introduction

The word “Novichok” entered the public consciousness through “the Salisbury poisonings” in March 2018 (Borger 2018; Philp 2018; Dodd et al. 2023). The term per se represents a relatively broad class of neurotoxic compounds. The group is listed among the organophosphorus (OP) compounds developed in the 1970s as a part of the Russian program Foliant (Mirzayanov 2008; Vásárhelyi and Földi 2007). The program was designed to produce new single components or binary agents from commonly available industrial chemicals. Such sources of chemicals should avoid raising suspicion of control organs. At the same time, emphasis was also placed on producing compounds more toxic and volatile than the existing Russian VX (a.k.a. VR, RVX, R-33, or VX) (Mirzayanov 2008). The Foliant program was supervised by Vladislav Gorodilov, who supposedly died from “Novichok” poisoning. After his death, the program was led by Peter Kirpichev, an official of the State Research Institute of Organic Chemistry and Technology (GOSNIIOKhT) in the Saratov region (Reiter and Gevorkyan 2018; Kanygin 2018; Sobchak 2020). The first information on these new OP compounds was announced by Vil Mirzayanov and Lev Fedorov in Russian newspapers at the beginning of the 1990s (quote) (Fedorov and Mirzayano 1992; Von Hippel 1993; Smithson et al. 1995).

“At the State Research Institute of Organic Chemistry and Technology (GOSNIIOKhT), a new chemical agent was created. In terms of its insidiousness (“combat characteristics”), it significantly exceeded the well-known VX; its damage is practically incurable. In any case, people who were once exposed to this chemical agent have remained disabled*.*”

A quote from the article “Poisoned Politics” first mentioned the research, production, and testing of chemical agents, weekly newspaper “Moscow News”, Sep 20, 1992, *#*38 (633) (Fedorov and Mirzayano 1992).

Interestingly, the name "Novichok" has never been officially included in the research program and appeared after the studies had been completed. The term refers only to binary agents that were effectively weaponized and tested. For example, Novichok-5 and -7 were binary agents synthesized from the base structure of A-232 and A-234, respectively (Mirzayanov 2008; Chai et al. 2018). This is in contrast with Pitschmann (2016), who misleadingly stated that “Novichok” is termed one of the subprojects of the Foliant special program. Hence, the term could have been allegedly used for the A-series compounds (non-binary) as well as their precursors and binary forms. Other details were uncovered in interviews with Vladimir Uglev, a prominent scientist involved in the secret research. Uglev claimed to be the co-author of A-232/Novichok-5 and confirmed the existence of its binary version (Waller 1997; Hoffman 1998). He also said that the name "Novichok" was common in the West only, but its Russian designers did not use it (Reiter and Gevorkyan 2018; Wintour 2018). Fedorov (1995) disputed the term "Novichok" in his book. Additionally, alleged “Novichok” poisoning survivor Vladimir Fedorov, a former GOSNIIOKhT technology section employee, confirmed the compound's code designation A-*xxx* (Sobchak 2020). Therefore, the designation A-series for these new compounds is seen as the only one correct. Based on the data collected, the Organization for the Prohibition of Chemical Weapons (OPCW) also concluded that "Novichok" should not be considered an independent group of chemical warfare agents. A-series compounds should be treated analogously to other nerve agents (NAs), possibly forming a separate group (Table 1) (Kloske and Witkiewicz 2019; Noga and Jurowski 2023).

**Table 1:** Generations of nerve agents

Nerve agent group	Examples
G-series	GA, GB, GD, GE, GF, GB-2
V-series	VX, RVX, VG, VX-2
GV-series^a^	GV
A-series	A-230, A-232, A-234, A-242, A-262

^a^Compound GV shares properties of both G- and V-series compounds. Overlapping GA and VA groups may emerge with advancing scientific knowledge

The structures of the A-series agents have never been officially disclosed. Several less efficient compounds developed during the Foliant program were systematically uncovered in the literature to hide the agenda of developing new NAs as a part of a pesticide research program (Kruglyak et al. 1972; Raevskiĭ et al. 1987a, b, 1990; Ivanov et al. 1990; Makhaeva et al. 1998). For the first time, the mass spectrum and structure of one of the compounds named "Novichok" were most likely recorded at the US National Institute of Standards and Technology (NIST) spectral database in 1998. The database indicated that the author of the Edgewood Center for Defense Research and Development of the United States Army provided the spectrum. This fact implies that this compound must have been synthesized in the USA and subjected to spectral analysis and possibly other research (VGTRK 2018; Executive Council 2018). Notably, the entry for this compound was deleted without any explanation in 2000. The head of the Russian Chemical Weapons Detection Laboratory revealed this fact on a Russian television program on Mar 25, 2018 (VGTRK 2018). He pointed out that Edgewood Arsenal had submitted a mass spectrometry profile for a compound called *N*-(*O*-ethyl-fluorophosphoryl)-*N',N''*-diethylacetamidine (corresponding to the structure designated A-234 in Mirzayanov's book). The NIST98 entry additionally referred to the Registry of Toxic Effects of Chemical Substances (RTECS) database, meaning the toxicity results were also submitted (VGTRK 2018; Executive Council 2018).

**Fig. 1:**
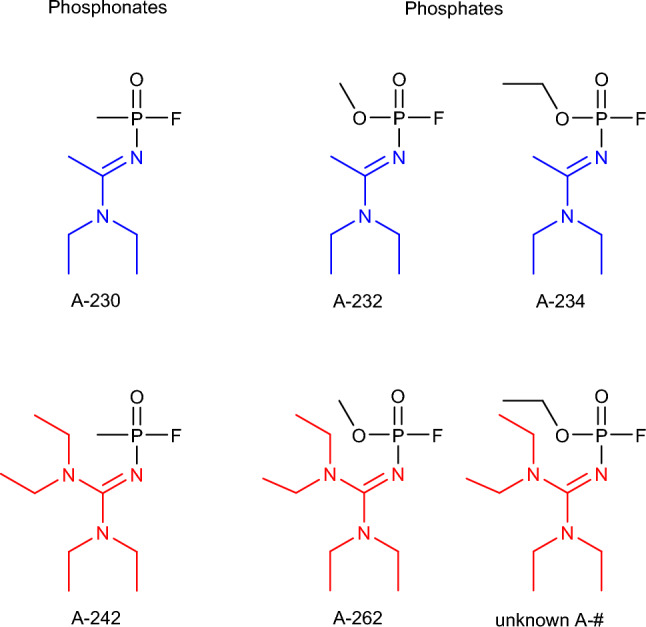
Molecular structure of A-series compounds proposed by Mirzayanov (Mirzayanov 2008) (color figure online)

The detective story continues. Mirzayanov (2008) disclosed the exact chemical structures of A-agents in his book. He rendered A-agents as phosphonamidofluoridate and phosphoramidofluoridate compounds (Fig. 1).

**Fig. 2:**
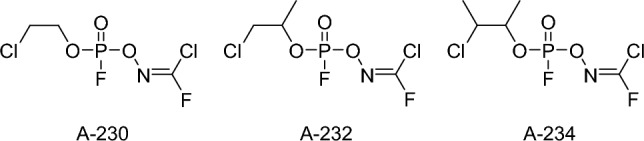
Different molecular structures of A-series published by Hoenig (2007) and Ellison (2008)

Mirzayanov noted that the synthesized unitary compounds and other OPs retained their original codenames (A-230, A-232, A-234, etc.). However, many alleged structures claiming to be "Novichoks" have been mentioned in the literature. For instance, Hoenig (2007) and Ellison (2008) published different formulas in 2007 (Fig. 2), citing a US Army source. According to these reports, the compounds contain carbonimidic alkyl monofluorophosphate substituents.

Professor Leonid Rink, another scientist who participated in implementing the Foliant program, claimed in 2018 that the structures published by Mirzayanov were the correct ones (Cockburn 2018; Wintour 2018). The phosphoramide formula also matched that in the Russian Ministry of Interior’s investigation report (Executive Council 2018; General Assembly Security Council 2018) and was indirectly verified by the US and Canada at the OPCW conference in November 2018. Both countries confirmed a match between Mirzayanov’s version of A-234 and the compound that poisoned the Skripals (Review Conference 2018a, b). However, A-series agents remain shrouded by mystery. The OPCW recognized only the compounds reported by Mirzayanov and included two new general structures and one individual compound into Schedule 1 on Dec 10, 2019, adjusting the Chemical Weapons Convention (CWC) Annex on Chemicals for the first time (Costanzi and Koblentz 2019). However, further modifications of CWC may follow. Constanzi and Koblentz (2021) noted that the joint proposal had not covered guanidine-bearing fluorophosphates such as A-262.

### Structures and synthesis of the a-series agents

**Fig. 3:**
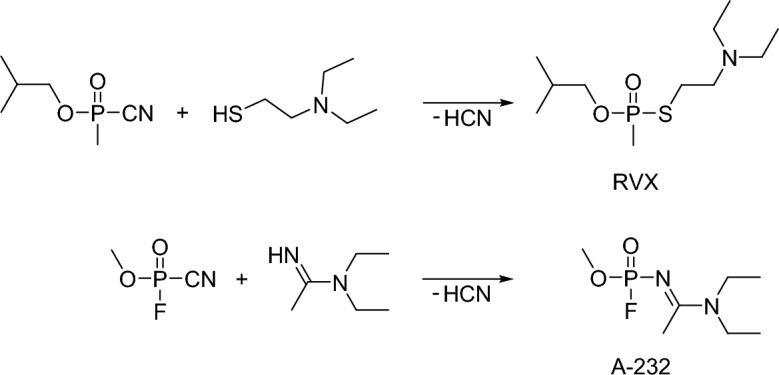
The synthesis of RVX and A-232 binary forms by Mirzayanov (2008)

A-series compounds have been initially derived from the V- and G-series agents (Smithson et al. 1995; Mirzayanov 2008; Halamek and Kobliha 2011; Kloske and Witkiewicz 2019; Costanzi and Koblentz 2021). Over a hundred analogs were allegedly synthesized and tested (Tucker 2006; Halamek and Kobliha 2011). Consistent with Mirzayanov, the typical nerve agent alkoxy substituent (–OR) on the central phosphorus atom is replaced in the case of A-agents by a nitrogen substituent (Pitschmann 2014; Franca et al. 2019). The first compound, substance-84/code designation A-230, is a sarin derivative, with an acetamidine moiety replacing the *O*-isopropyl group. After A-230, Peter Kirpichev and his group synthesized and tested A-232, A-234, A-242, and A-262 (Mirzayanov 2008). Notably, most A-series agents follow the A-230 design. A-232 and A-234 structures are A-230 methoxy and ethoxy analogs, respectively, with acetamidine moieties (Fig. 1, blue color). Other analogs, namely, A-242 and A-262, are guanidine analogs (Fig. 1, red color). Agents A-230 and A-242 belong to the group of phosphonates, while agents A-232, A-234, and A-262 are phosphates (Costanzi and Koblentz 2021). In his book, Mirzayanov (2008) published a simple schematic synthesis of A-234 from direct binary precursors based on the route used to provide V- and G-series agents (Fig. 3).

**Fig. 4:**
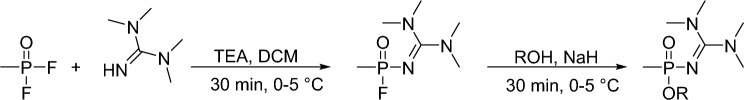
The final structure shown is *O*-alkyl *N*-(bis(dimethylamino)methylidene)-*P*-methylphosphonamidate, and the intermediate has a bis(dimethylamino)methylene moiety; *DCM* dichloromethane, *TEA* triethylamine; according to (Hosseini et al. 2016, 2021; Eskandari et al. 2022; Carvalho-Silva et al. 2023; Noga and Jurowski 2023)

Still, the discussion on the synthesis must be cautious due to many conflicting structures, potential misuse, and extraordinarily little information available in the literature. Many authors refer only to Mirzayanov and add proposed chemistry schemes of synthesis without any detailed information on the synthesis-specific conditions (Halamek and Kobliha 2011; Chai et al. 2018; Nepovimova and Kuca 2018; Kloske and Witkiewicz 2019; Franca et al. 2019; Vicar et al. 2021). The exception can be found in the work of Hosseini and colleagues, publishing a study related to this topic in 2016. Iranian scientists prepared five A-agent derivatives using micro-scale conditions (Hosseini et al. 2016). However, none of their structures precisely matches the A-series compounds Mirzayanov uncovered. The structures reported by Hosseini et al. (2016) were close to A-242. The only difference was the presence of methyl substituents on the nitrogen atom instead of ethyl groups. The synthetic approach of this compound was feasible by the controlled reaction of methylphosphonyl difluoride and *N,N,N',N'*-tetraethylguanidine or *N,N,N',N'*-tetramethylguanidine (Fig. 4).

The same group released two more studies related to chemistry. They described the microsynthesis of selenophosphorus compounds as close analogs to A-series agents in 2021 (Hosseini et al. 2021). A year later, they disclosed new data on synthesizing A-230, A-232, A-234, and six other compounds using previously published procedure (Eskandari et al. 2022). Based on the work of Hosseini et al. (2016) and another publication (Ledgard 2006), it is possible to deduce the synthesis route of compounds reported by Mirzayanov from commercially available building blocks. All other studies working with A-series agents continue to bypass the publishing of their procedures by just referring to a military provider or unspecified "in-house" methods. The Brazilian group reported a reaction scheme similar to that of the Iranian group for the microsynthesis of the A-242 analog (Carvalho-Silva et al. 2023).

### Physicochemical properties of A-series agents

The availability of physicochemical properties information on A-series from public sources is scarce. Mirzayanov (2008) reported only limited data. According to his book, A-230, A-232, and A-234 are liquids. A-230 crystallizes at temperatures below − 10 °C. A-232 and A-234 are more stable, so they can be used in winter, unlike the A-230 variant (without the solvent *N,N*-dimethylformamide to prevent crystallization). A-232 possesses higher volatility but is less stable to moisture than A-230 (and VX). Agents A-242 and A-262 should be solids. Mirzayanov (2008) also estimated the hydrolysis half-life of A-234 at pH 6.5*–*7.4 to be moderate, i.e., 10–30 days. Several studies describing A-agent properties only referred to Mirzayanov's observations (Halamek and Kobliha 2011; Pitschmann 2014).

Nepovimova and Kuca (2018) published a complex overview of A-series agent properties, including boiling point, density, state, behavior at low temperatures, volatility, and moisture stability. However, the review referred to unsubstantiated data and, for instance, speculated on the low environmental stability. And notably, the facts from the Salisbury poisoning investigation suggest the opposite (Peplow 2018).

Similarly, Franca et al. (2019) estimated melting point, boiling point, vapor pressure, and solubility in their review a year later. In the same study, logP was calculated, indicating a high lipophilic character of the compounds, contributing to high permeation into the body. Other molecular modeling studies discussed various parameters, covering different structural, electric, spectral, thermodynamic, thermal, and hydrolytic properties (reviewed in Table 2). These results represent a helpful starting point for laboratory experiments, which should validate them. For instance, Bhakhoa et al. (2019) modeled hydrolysis under neutral conditions (among other parameters). They identified two active electronegative centers (phosphorus atom/P_4_ and the hybridized carbon atom/sp^2^), with fragmentation being more probable on the carbon atom. As another example, Otsuka and Miyaguchi (2021) calculated hydrolysis in an alkaline environment, indicating that such conditions would yield easier hydrolysis than neutral conditions. Both studies were later contradicted by experimental work by Lee et al. (2021). Thus, the hydrolytic degradation calculated based on the reaction mechanism and activation energy may not always be reliable. Yet, effective hydrolysis is significant, especially when dealing with decontamination.

**Table 2:** Studies modeling various A-series agents´ properties

Authors	A-series agent	Modeled properties	Outcomes
Franca et al. (2019)	A-230, A-232, A-234	logP	Strong lipophilic character
Carlsen (2019)	A-230, A-232, A-234, A-242, A-262	Vapor pressure, hydrolysis	Agents A-230, A-232, and A-234 have higher vapor pressures, while A-242 and A-262 possess lower vapor pressures than VX. All agents are defined with slow hydrolysis and biodegradation
Lyagin and Efremenko (2019)	A-232	Enzyme-catalyzed hydrolysis	Organophosphate hydrolase may degrade the agent
Bhakhoa et al. (2019)	A-234	Molecular, electronic, spectroscopic, thermodynamic properties, potential thermal, and hydrolysis degradation	Enthalpy and energy changes in hydrolysis and solvolysis of A-234. The electropositive charge on the phosphorus atom (P_4_) and the hybridized carbon atom (sp^2^) suggest two possible hydrolytic pathways. Higher probability for the carbon atom
Tan et al. (2019)	A-232	Electronic properties, vibrational spectra	Ultra-sensitive detection of the novel agent A-232 by vibrational spectroscopy
De Farias (2019)	A-234	Molecular, electronic properties	Both substances exhibit a smaller number of conformers and a higher dipole moment compared to VX. That explains why these substances are as toxic as VX
Nakano et al. (2019)	A-230, A-232, A-234	Absorption spectra of neutral specie and singly charged ion	The A-series molecules can be ionized. The wavelengths for the first excited energy, the ionization energy, and the half-ionization energy have been calculated
Imrit et al. (2020)	A-234	Hydrolysis and fragmentation	Possible hydrolysis of side chains under neutral conditions. Substitution attack by a water molecule on the acetamidine branch is thermodynamically more efficient than substitution on the central phosphorus of the molecule
Motlagh et al. (2020)	A-234	Electronic properties, adsorption energies, fullerene capacity	The adsorption energies of A-234 are very high. A suitable nanosensor base for detecting the A-234 complex of C_20_ fullerene molecule (C_20_HNH_2_)
Yar et al. (2021)	A-230, A-232, A-234	Adsorption and electronic properties of analytes on the carbon nitride 2-D (C2N) surface	Prediction of interaction between analytes and C2N surface for electrochemical detection
Otsuka and Miyaguchi (2021)	A-230, A-232, A-234	Hydrolysis and fragmentation	A-230 is more easily hydrolyzed than A-232 and A-234. A-series agents are similar to VX but more hydrolysis-resistant than GB under basic conditions, which is better than neutral conditions for efficient decontamination. The activation energy of A-234 hydrolysis under alkaline conditions is smaller than all others. Fluorine release occurs more quickly than acetamide release in A-agents
Vieira et al. (2021)	A-230, A-232, A-242, A-262	Structural, electronic, and thermodynamic properties, spectroscopic parameters	A-series molecules have two electropositive centers. LogP values confirm high lipophilicity (but less than VX). The central phosphorus atom (P_4_) is more positively charged than the hybridized carbon atom (sp^2^). Therefore, they preferentially accept electrons in the chemical reaction and form a bond with the nucleophile S_N_2
Chernicharo et al. (2021)	A-230, A-232, A-234	Comprehensive analysis of fragmentation pathways	The expected secondary fragmentations have been identified, such as the elimination of fluorine on the phosphorus atom and the formation of the acetamidine chain
Sajid et al. (2021)	A-230, A-232, A-234	Electronic properties, adsorption energies	Stability of A-agents and graphdiyne complexes (GDY). The adsorption energy of A-234-GDY > A-232–GDY > A-230-GDY
Eskandari et al. (2022)	A-230, A-232, A-234, and other analogs	Structural, electronic, and thermodynamic properties, retention, and electrophilicity indices	The central phosphorus atom is more positive and thus reacts with the nucleophile S_N_2. Measured mass fragmentation pathways. Simulated IR and NMR data of agents A-230 and A-232
Jeong et al. (2022a)	A-230, A-232, A-234	Kappa, molecular weight, hydrogen bond acceptor, the complexity of bonding and distribution of heteroatoms, hydrogen bond donor, TPSA, logP, vapor pressure	Provided calculated values of the mentioned parameters, with logP confirming the highest lipophilicity of A-234
Kim et al. (2022)	A-230, A-232, A-234	Spectroscopic parameters (IR spectra)	Predicted high-accuracy IR spectra of A three A-series agents
Jeong et al. (2022b)	A-232, A-234	Nuclear magnetic resonance spectra	^1^H and ^13^C NMR prediction for 83 A-series candidates, which were experimentally confirmed for A-232 and A-234
Rashid et al. (2023)	A-230, A-232, A-234	Electronic structures properties (electrophilicity index) and hydrolysis rate	The hydrolysis rate of A-series is lower than that of V-series nerve paralyzers and significantly lower than that of G-series. They compared the experimental hydrolysis rate data with the prediction hydrolysis rate data calculated using the electrophilic index. The trend of the hydrolysis rate between A-230 > A-232 > A-234 corresponded with the lipophilicity of molecule A-234 > A-232 > A-230
Noga et al. (2023a)	A-230, A-232, A-234, A-242, A-262	Hydrolysis and biodegradation	Evaluation of hydrolysis estimates showed extremely rapid degradation of compounds A-230 and A-242 in contrast to A-232, A-234, and A-262
ChemSpider (b, 2018c, 2018a)	A-230, A-232, A-234	Density, boiling point, vapor pressure, enthalpy, flash point, etc.	Predicted by ACD/Labs ChemAxon

Means of modeling: Franca et al. (2019)—chemicalize.com; Carlsen et al. (2019)—QSAR modeling; Lyagin and Efremenko (2019)—molecular docking; Bhakhoa et al. (2019)—DFT; Tan et al. (2019)—DFT; De Farias (2019)—SE method; Nakano et al. (2019)—DFT; Imrit et al. (2020)—DFT; Motlagh et al. (2020)—DFT; Yar et al. (2021)—DFT; Otsuka and Miyaguchi (2021)—DFT; Vieira et al. (2021)—DFT, QSAR modeling; Chernicharo et al. (2021)—DFT; Sajid et al. (2021)—DFT; Eskandari et al. (2022)—DFT; Jeong et al. (2022a)—DFT, QSAR modeling; Kim et al. (2022)—DFT; Jeong et al. (2022b)—DFT; Rashid et al. (2023)—DFT; Noga et al. (2023a, b)—QSAR modeling

*DFT* density functional theory, *SE* semiempirical, *QSAR* quantitative structure–activity relationship, *n/a* not available

Experimental data on the physical and chemical properties of A-agents are limited to spontaneous hydrolysis, degradation in acidic or alkaline conditions, and enzymatic degradation. Harvey et al. (2020) published the first results on some A-series agents' stability and hydrolysis rate in 2020. The study showed that the hydrolysis was 2–3 and 0–2 orders of magnitude slower than for the G- and V-series agents, respectively, confirming the stability of A-series agents in the environment. The study also disclosed the activation energies for A-230, A-232, and A-234 and kinetic values for organophosphorus acid anhydrolase (OPAA) hydrolysis, resulting in 2*–*3 orders lower magnitude than of the G-series agents and 2 orders higher magnitude than of the V-agents. Nevertheless, their results were based on a 10-min measurement, evaluating only released fluorides.

**Fig. 5:**
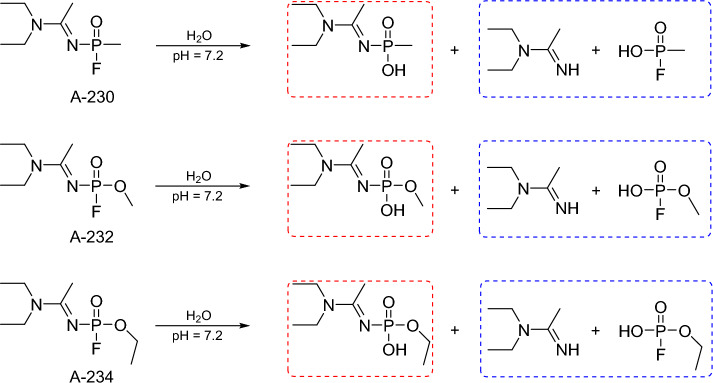
Hydrolytic pathways of A-230, A-232 and A-234 with major (red frames) and minor (blue frames) products under neutral conditions (Lee et al. 2021; Jacquet et al. 2021; Otsuka and Miyaguchi 2021; de Koning et al. 2022; Jung et al. 2023; Noga and Jurowski 2023) (color figure online)

Lee et al. (2021) studied the degradation modes of A-234 under three different pH conditions (pH 3.5, 7.2, 9.4). Regardless of pH, the main fragmentation product was ethylhydrogen (1-(diethylamino)ethylidene)phosphoramidate, while *N,N*-diethylethanimidamide and ethylhydrogenphosphorofluoridate were minor products (Fig. 5). It implies that the phosphorus atom can be designated as the hotspot for degradation, being the major active electropositive center.

The study also showed hydrolysis of A-agents was more effective under acidic conditions than neutral or alkaline ones. Such a phenomenon was explained by the increased positive partial charge of the phosphorus atom due to the easily protonated nitrogen atom of the acetamidine group at low pH of 3.5 (Lee et al. 2021). The study aimed to simulate natural environmental conditions, avoiding solutions with extremely low or high pH values. De Koning et al. (2022) confirmed low stability of A-230 in pH 4.5 because it was completely degraded within 16 h. A-232 and A-234 appeared stable. However, their hydrolysis in acidic, neutral, and alkaline conditions (pH 10) was measured only for 1 h. Similarly, Jacquet et al. (2021) confirmed the high stability of A-230, A-232, and A-234 in pH 9.5, conducting only a 1-h study.

In contrast, Jung et al. (2023) focused on degradation by strong acids and bases such as NaOH and HCl for decontamination. They confirmed that extreme alkaline and acid solutions could effectively hydrolyze A-232 and A-234. The stabilities of A-series agents derived from different experiments are summarized in Table 3.

**Table 3:** Stability and hydrolysis of A-series compounds

Study	Buffer, pH	Reaction conditions (temperature, time)	Results
Harvey et al. (2020)	BTP, 7.2	25 °C, 10 min	A-230, A-232, and A-234 stable for 10 min
Lee et al. (2021)	AA (aq)^a^, 3.5	n/a °C, 1 week	A-234 completely degraded after a week
	DIW, 7.2	n/a °C, 2 months	A-234 completely degraded after 2 months
	PC_(aq)_^b^, 9.4	n/a °C, 2 months	A 234 remained stable after 2 months
de Koning et al. (2022)	TBS, 4.5	25 °C, 30 h (A-230); 1 h (A-232, A-234)	A-230 completely degraded in 16 hA-232 and A-234 are stable for 60 min
	TBS, 7	25 °C, 1 h	A-230, A-232, and A-234 stable for 60 min
	TBS, 10	25 °C, 1 h	A-230, A-232, and A-234 stable for 60 min
Jacquet et al. (2021)	TBS, 9.5	25 °C, 1 h	A-230, A-232, and A-234 stable for 60 min
Jung et al. (2023)	DIW, 7.2	20–25 °C, 1 h	A-232 and A-234 stable for 60 min
	HCl, 1	30 min	A-232 and A-234 completely degraded
	NaOH, 13	30 min	A-232 and A-234 completely degraded

*BTP* BIS–TRIS propane, *TBS* TRIS-buffered saline, *DIW* deionized water, *n/a* not available

^a^0.01 M AA_(aq)_, acetic acid aqueous solution

^b^0.01 M PC_(aq)_, potassium carbonate aqueous solution

Jacquet et al. (2021) additionally tested the catalytic effectivity of two engineered phosphotriesterase (PTE) enzymes developed for rapid hydrolytic detoxification of OPs. Both enzymes could degrade all three agents, with the mixture of the two enzymes being the most effective. Interestingly, A-232 and A-234 hydrolysis was a one-step reaction, while the hydrolysis of A-230 underwent two steps. Later, de Koning et al. (2022) effectively decomposed A-agents using the MOF-808 Zr metal–organic framework under alkaline conditions. This material is a highly efficient and regenerative catalyst, and the degradation was carried out in two steps. The initial degradation rate of A-230 and A-232 was fast, while considerably slower in the case of A-234. Catalytic degradation is summarized in Table 4.

**Table 4:** Catalytic degradation of A-series substances

	Enzyme(s)						Zr MOF-808 (mol 6%) (de Koning et al. 2022)		
	Modified PTE (Jacquet et al. 2021)			Wild-type OPAA (Harvey et al. 2020)					
NAs	A-230	A-232	A-234	A-230	A-232	A-234	A-230	A-232	A-234
t_1/2_ (min)	< 0.8	2.5	0.8	n/a	n/a	n/a	0.094	0.42	7.3
K_cat_ (min^–1^)	> 312	222 ± 6	> 312	870 ± 52	900 ± 151	47 ± 74	7.35 ± 1	1.67 ± 0.1	0.10

*n/a* not available

### Mechanism of action

The symptoms of intoxication and the effectiveness of anticholinergics confirm that the A-series mechanism of action is associated with acetylcholinesterase (AChE, E.C. 3.1.1.7) inhibition. The first data based on modeling started to appear after the Salisbury incident. Carlsen (2019) assessed the probability of cholinomimetic effects using the Prediction of Activity Spectra for Substance (PASS) prediction tool. All five numbered A-series compounds were positive for this biological activity. However, such prediction is highly limited by using only 2D structure for the calculation and not including molecular energy levels (Parasuraman 2011). Bhakhoa et al. (2019) modeled the reaction between A-234 and the AChE enzyme. The study utilized methanol as the simplest model for the active serine site of the enzyme, which is burdened by several limitations. Jeong and Choi (2019) performed a thermodynamic study, but they evaluated the reaction of serine with the compounds proposed by Hoenig (2007) and Ellison (2008) only. A research group from the University of California, San Diego, published the most relevant information on the interaction between AChE and A-series molecules. Luedtke et al. (2021) and Radić (2021a, 2023) used X-ray structural data of recombinant human AChE (hAChE) inhibited by A-234 uploaded into the Protein Data Bank. They conducted a computational study of different OP–hAChE conjugates, including those of A-230, A-232, and A-234. They pinpointed that even bulkier structures like A-series agents fit into the hAChE active site without significant steric hindrance onto the hAChE backbone or side chains and can form stabilizing hydrophobic or electrostatic interactions with the choline-binding site. Such stabilization could render them more resistant to nucleophilic reactivation with oxime antidotes (Blumenthal et al. 2021; Luedtke et al. 2021; Radić 2021, 2023). They also presented A-234–hAChE conjugate very vividly in virtual reality on YouTube (Radic 2021b).

**Fig. 6:**
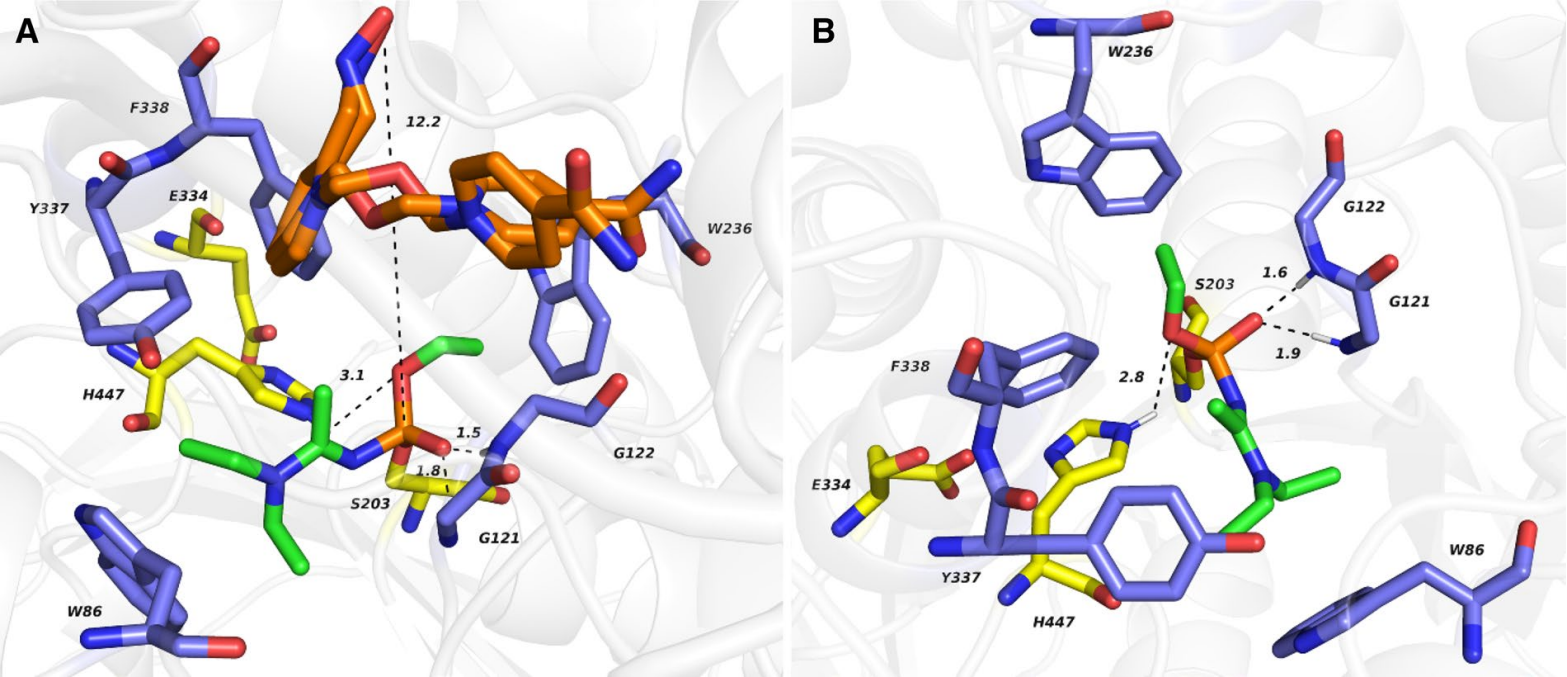
Crystal structure of the hAChE–A-234-inhibited complex with oxime reactivator HI-6 (**A**; PDB ID: 6NTG) and hAChE–A-234-inhibited adduct (**B**; PDB ID: 6NTL). A-234 is rendered by green carbon atoms, catalytic triad residues by yellow carbon atoms, and essential amino acid residues involved in interacting with the A-234 agent in blue carbon atoms. The oxime reactivator is displayed in orange (**A**), captured in two orientations, and stacked at the enzyme’s peripheral anionic site. The hydrogen bond interactions are shown as dashed lines with distances measured in Å. Figures were created by Pymol v. 2.4.1. (Bester et al. 2020c, d) (color figure online)

Crystal structures of hAChE inhibited by A-230, A-232, and A-234 in complex with the HI-6 reactivator are now available in the Protein Data Bank (Bester et al. 2020a, b, c, d). The distance between the oxime reactivation warhead of HI-6 and the central phosphorus atom of A-234 is 12.2 Å (PDB ID: 6NTG), implying no possibility for a nucleophilic attack and thus no or negligible reactivation ability of HI-6 (Fig. 6A). The similar distance between the phosphorus atom of A-230 and the oxime moiety of HI-6 (PDB ID: 6NTN), and A-232 and the oxime moiety of HI-6 (PDB ID: 6NTM) equal 10.1 and 10.0 Å, respectively, also presuming no reactivation process (not shown). For clarity, we also provide the hAChE–A-234-inhibited complex (Fig. 6B).

Only one study has established the analytical data on the reactivation and aging of A-agent-inhibited AChE, using only an A-242 surrogate and non-human AChE. Santos et al. (2022a) synthesized *N,N,N',N'*-tetraethyl-*N*''-(methyl(4-nitrophenoxy)phosphoryl)guanidine and evaluated the efficacy of pralidoxime, trimedoxime, obidoxime, and HI-6 to reactivate the surrogate-inhibited Electrophorus eel AChE in vitro. The study showed no aging after 90 min of enzyme inhibition and highlighted trimedoxime as the most promising oxime. The finding had been supported by modeling (Santos et al. 2022b).

Finally, no relevant information about interactions with other proteins, including butyrylcholinesterase (BChE, E.C. 3.1.1.8), is available. However, the A-series amine group may interact with different targets, widening the spectrum of action mechanisms that could affect the clinical picture of toxidrome.

### Toxicity

Information on the toxicity of A-series compounds remains highly elusive. Mirzayanov (2008) described that the toxicity of Novichok-5 was 5–⁠8 times higher than that of VR, while Novichok-7 was 10 times more potent than soman. He also noticed that compounds A-242 and A-262 should be highly toxic. Earlier in 1997, Bill Gertz (1997) stated that A-232 and A-234 are "as toxic as VX, as resistant to treatment as soman, and more difficult to detect and easier to manufacture than VX," referring to a classified report by the US Army’s National Ground Intelligence Center.

Many authors have speculated about the toxic properties of the A-agent or referred to unpublished information (Hoenig 2007; Karev 2009; Nepovimova and Kuca 2018; Franca et al. 2019; Carlsen 2019). According to Karev (2009), finding accurate toxicological data from Russian sources is possible. However, his seminal paper did not provide references. Franca et al. (2019) attempted to estimate LCt_50_ and LD_50_, i.e., lethal concentration in the environment and lethal doses, based on Mirzayanov and other literature. However, the study mismatched the compounds disclosed by Mirzayanov to those depicted by Hoenig (2007) and Ellison (2008). Carlsen (2019) contested Mirzayanov’s statement using computer modeling. Their study calculated the lethal doses (LD_50_) of five A-series compounds for the oral exposure route in rats using the Toxicity Estimation Software Tool (TEST). These data were then translated into humans. The prediction showed that the toxicity of the A-series was 5–75 times lower than that of VX. Noga et al. (2023b) conducted a similar in silico acute toxicity study of reagent A. They calculated the average *per oral* lethal doses for rats utilizing two software tools, the quantitative structure–activity relationship (QSAR) Toolbox and the TEST Consensus method, and then they extrapolated animal data to humans. The lethal toxicity was predicted as follows: A-232 > A-230 > A-234 > A-242 > A-262 (summarized in Table 5).

**Table 5:** Available data on the toxicity of the A-series agents

NAs	VX	A-230	A-232	A-234	A-242	A-262	Study
LD_50_ (mg·kg^–1^)	0.14	n/a	0.01–0.03	0.07	n/a	n/a	Karev (2009)^a^
	0.09–0.14	0.1–0.03	0.5	0.5	n/a	n/a	Ellison (2017)^b^
	10	n/a	1–2	5	n/a	n/a	Nepovimova and Kuca (2018)^c^
	0.1	0.01–0.03	0.5	0.5	n/a	n/a	Franca et al. (2019)^c^
	0.10	1.55	0.57	0.71	0.49	7.35	Carlsen (2019)^d^
	n/a	0.50	0.41	0.63	9.02	22.85	Noga et al. (2023b)^d^
	n/a	0.35	0.21	0.58	14.97	44.56	Noga et al. (2023b)^e^
LCt_50_ (mg·min·m^–3^)	50	n/a	6–10	7	n/a	n/a	Nepovimova and Kuca (2018)^c^
	15	2–3	7	7	n/a	n/a	Franca et al. (2019)^*c*^

*n/a* not available

^a^According to Russian literature (did not specify sources)

^b^Unknown source

^c^Used Karev’s and Ellison’s data

^d^QSAR Toolbox, quantitative structure–activity relationship

^e^TEST Consensus method, Toxicity Estimation Software Tool

De Farias (2019) and Jeong et al. (2022a) published different results, although both studies did not provide specific values. De Farias (2019) evaluated the toxicity parameters for A-230 and A-234 using DFT calculations. He concluded that fewer conformers with high dipole moments could be associated with a higher biological/toxic activity, comparing the toxicity of both compounds to VX. Jeong et al. (2022a) defined a different order of toxicity, with A-234 being the most poisonous, followed by A-232, and A-230 designated as the least toxic. They also perceived propyl-bearing derivatives from all the A-agents as less toxic. Importantly, we must remember that acute oral OP toxicity in mammals correlates poorly with enzyme inhibitory activity, implying that toxicity cannot be assessed only by computational data (Wang et al. 2021; Bolt and Hengstler 2022). So far, no experimental data on A-agent toxicity have been published.

Publicly available experimental data on symptoms of poisoning are now almost non-existent. In 2019, the U.S. government published medical management guidelines on the A-series agents, claiming bronchoconstriction and seizure activity had been a prominent feature of their toxicity in animals (Chemical hazards emergency medical management (CHEMM) (2019). Nevertheless, other experimental data remained classified. Exposed victims represent another valuable source of information. These sources include one original article, police reports, and direct interviews with victims in the newspaper. We could find five confirmed or highly probable incidents involving A-series agents. The first victim of intoxication was a scientist working in the Foliant program. Other events can be classified as deliberate poisonings of four people. Besides them, seven other victims displayed significant signs of intoxication. More than 14 other people were possibly exposed to A-agents, but they either showed minor symptoms or no data were given. Each case of A-agent intoxication is listed in Table 6. Newspaper articles also indicate that there may have been more incidents. For instance, they suggest field accidents, testing new OP compounds on soldiers, or a link to the assassinations of Muslim Chechen leaders in 2002 (Ibn al-Khattab) and 2013 (Dokka Umarov). However, no solid evidence exists (Nepovimova and Kuca 2018; Rozhdestvensky 2018; Knight 2018, TV Rain 2021; Dzutsati 2021).

**Table 6:** Victims of A-series agent poisoning

Year of incident	Victim (age)	Route of exposure	Onset	Acute symptoms	Hospitalization	Therapy	Outcome	Possible delayed symptoms
1987^a^ (Tucker 2006; Mirzayanov 2008; Dobrynin 2018; Roth and McCarthy 2018)	Man (n/a)	Inhalation	Immediately	Mydriasis, bronchorrhea, vomiting, hallucinations, unconscious	Over 1 week	Vodka, tea, atropine	Survived	Inability to walk, read, and concentrate, chronic arm weakness, trigeminal neuritis, hepatitis with subsequent cirrhosis, epilepsy, depression
1995^b^ (110–114) (Stanley 1995; Kislinskaya 2001; Felshtinsky and Pribylovsky 2010; Shleinov 2018a, b)	Man (46)	Transdermal, inhalation exposure was also possible	Hours: the police report states that the latent phase for skin exposure could last 1.5–5 h	Complete picture NA (coma, multiorgan failure)	3 days	n/a, possibly symptomatic, except for small doses of atropine for examination of the eye fundus	Dead	n/d
	Woman (35)			Full picture NA (convulsions, circulatory disorder, coma, multiorgan failure)	1 day	n/a, possibly symptomatic	Dead	n/d
				Other victims complained about headaches, dizziness, lacrimation	n/a	n/a	n/d	n/d
2015^c^ (115–118) (Bellingcat Investigation Team 2019a, b; Dimitrov 2019; The Insider and Bellingcat 2020)	Man (65)	Transdermal, inhalation exposure was also possible	Hours	Nausea, vomiting, eye itching, vision disorders, coma	17 days	n/a	Survived	n/a
	Man (n/a)		Hours	Vomiting, slurred speech, blurred vision, miosis, excessive sweating, hypertension, coma	9 days	n/a	Survived	n/a
	Man (n/a)		Four days	Full picture NA (milder than the two previous victims, coma)	n/a	n/a	Survived	n/a
2018^d^ (Stone 2018; BBC News 2018a, b; Technical Secretariat 2018; PHE 2018; Technical secretariat 2018; Counter Terrorism Policing 2018; Vale et al. 2018; Morris et al. 2019; Ridley 2019; Morris 2021)	Man (66)	Transdermal	Hours	Briefing note released by PHE summarized symptoms: painful dim vision, miosis, involuntary defecation, impaired breathing, sinus bradycardia, fasciculations, muscle weakness, hypotension, convulsions, coma	72 days	Atropine, pralidoxime, ventilation, neuroprotection, and probably also naloxone to exclude opioid overdose	Survived	n/a
	Woman (33)				37 days		Survived	n/a
	Man (41)				16 days		Survived	n/a
	Man (45)				18 days		Survived	n/a
	Woman (44)	Transdermal, inhalation exposure was also possible	Minutes		8 days		Dead	n/d
2020^e^ (Science’s news staff 2020; Smolentseva 2020; Stone 2020; Technical Secretariat 2020; Steindl et al. 2021)	Man (44)	Transdermal	Hours	Nausea, vomiting, hypersalivation, sweating, miosis, conjunctival injection, bradycardia, collapse, muscle stiffness, convulsions, hypothermia, confusion, unconsciousness, the elevation of plasma lipase and amylase	31 days	Atropine, obidoxime, midazolam, fentanyl, tropicamide, morphine, propofol, crystalloids, ventilation	Survived	n/a

*PHE* public health England, *n/a* not applicable, *n/d* no data

^a^The first known victim of poisoning with “Novichok,” specifically A-232, was scientist Andrei Zheleznyakov in 1987 after the ventilation in his laboratory failed (Mirzayanov 2008; Roth and McCarthy 2018)

^b^Russian banker Ivan Kivanli and his secretary were intoxicated with a military-grade nerve agent (Stanley 1995). The exposure (A-234, according to the chromatograph in the police report) most likely occurred through contact with the telephone receiver. The police file mentions that signs of intoxication were also present in his bodyguard, visitors, a cleaning lady, and 8 police officers (Shleinov 2018a, b)

^c^Bulgarian arms dealer Emilian Gebrew and a close business partner were exposed to unknown OP through contact with car door handles. Gebrev’s son was also intoxicated. Newspapers state that two other men fell ill, but symptoms were not mentioned. The Finnish laboratory VERIFIN found traces of two organophosphorus compounds in a blood sample (Bellingcat Investigation Team 2019a). Following the publication of the poisoning of Sergei Skripal, the same compound (A-234) was suspected (Bellingcat Investigation Team 2019b). The loss of the tested samples made it impossible to identify the exact composition of the agent (Morris et al. 2019)

^d^A former GRU agent, Sergei Skripal, and his daughter were intoxicated with A-234 (Security Council 2018). Four months after their poisoning, two other citizens were exposed to the same substance found in a vial (Ridley 2019). The UK government’s Defence Science and Technology Laboratory (DSTL) at Porton Down confirmed the same identity of the substance (Technical secretariat 2018). Another 22 people were investigated for intoxication, with 1 showing mild symptoms

^e^Poisoning of Alexei Navalny. The detected biomarkers in the samples indicated a toxic substance structurally, like A-series nerve agents (Technical Secretariat 2020). This phosphate compound carried a guanidine moiety, mimicking A-242. The substance used for poisoning was possibly A-262 or another closely related phosphate substance bearing the same branch (Technical Secretariat 2020)

The cases in Table 6 indicate that the typical A-agent exposure route was transdermal intoxication. Such a route could be perceived as desired in assassinations for slowing the onset of symptoms. Inhalation was the primary route of exposure in the first victim reported in 1987. He was accidentally exposed when a hood vent malfunctioned, releasing a small amount of concentrated A-232 into the air. Oral poisoning did not play a role in the presented cases, although Mirzayanov emphasized that ingestion and inhalation would be the most probable routes of poisoning (BBC News 2020).

Several factors influence the onset of OP intoxications, including dose, exposure time, route of poisoning, and therapeutic intervention. (Marrs et al. 2007; Ciottone 2018; Costanzi et al. 2018). The inhalation route is associated with the rapid onset of symptoms. According to a briefing note released by Public Health England, toxidrome develops within minutes to hours after exposure, usually less than 6 h. The note also recommends investigating any illness occurring within 12 h after potential contact with suspect material or contaminated location (PHE 2018). On the other hand, low-dose exposure may have a latency of up to several days, as shown in a victim from 2015. Low-dose exposure most likely played a significant role in this case.

The symptoms exhibited by the cases have been typical of OP poisoning arising from overstimulation of muscarinic and nicotinic receptors. Clinicians usually refer to DUMBLES abbreviating defecation/diaphoresis, urination, miosis, bronchospasm and bronchorrhea, lacrimation, emesis, and salivation. Another term, SLUDGE, covers only "wet signs," including salivation, lacrimation, urination, diaphoresis, gastrointestinal discomfort, and emesis (Saalbach 2023). Severe hypothermia was reported in one victim intoxicated in 2020 (Steindl et al. 2021). However, the symptom can be seen in up to 50% of OP poisonings (Moffatt et al. 2010; Mozafari et al. 2016; Wang et al. 2021). Mydriasis was observed only in the first victim, possibly developed upon prevailing nicotinic receptor overstimulation. The briefing note reviewing the Salisbury and Amesbury incidents emphasizes that blurred vision with either miosis or mydriasis is the best descriptor. The death of the intoxicated victims occurred between 1 to 8 days after exposure. In surviving patients, hospitalization took approximately 29 days (16–72 days). But, the small number of casualties and lack of information about the agent and the dose used do not allow for drawing any clear conclusions. The long-term prognosis is similarly uncertain because most of the incidents are recent. However, the victim from 1987 reported disabling neurological and neuropsychological symptoms, indicating that A-series agents may cause delayed neuropathy (Mirzayanov 2008; Noga and Jurowski 2023).

### Toxicokinetics

Reliable information on toxicokinetics is also minimal. Incidents involving A-series agents verify inhalation and transdermal routes of intoxication. The calculated partition coefficient and vapor pressure mentioned above support both observations (Bhakhoa et al. 2019; Franca et al. 2019; Carlsen 2019; Vieira et al. 2021; Jeong et al. 2022a). Bhakhoa et al. (2019) and Carlsen (2019) provided more detailed computational models. Bhakhoa et al. (2019) modeled the lipophilicity, solubility, topological polar surface area, and skin permeability of A-234 using the SwissADME tool. They indicated high human gastrointestinal absorption and good skin and blood–brain barrier permeability. However, they assessed A-234 as less skin permeant than VX but still crossing the barriers quickly. Carlsen (2019) used various QSAR models, including the finite-dose skin permeation calculator and the ACD/Percepta platform, to estimate skin permeation, first-pass metabolism after oral administration, oral bioavailability, time for maximum plasma concentration, elimination rate constant, and elimination half-life. They predicted slower human skin permeation and a reduced amount of all five numbered A-series agents permeating human skin compared to VX. They also noted that slower skin permeation could lead to a prolonged recovery. First-pass metabolism after oral administration was estimated at approximately 35–50%, the time for maximum plasma concentration was about 55–70 min, and elimination half-lives were 3.5–4 h (comparable to VX). Nevertheless, published animal data confirming such calculations do not exist.

### Diagnostics and retrospective detection

Detection and biomonitoring currently represent the most robust lines of research on A-series agents (Bolt and Hengstler 2022). The specific determination of the poison is essential for diagnosis and selecting adequate therapy. Considering the laboratory capabilities of current hospitals and the acuteness of poisoning progression, doctors could only diagnose non-specific cholinesterase inhibition based on the developing cholinergic toxidrome and decreased BChE levels in the patient´s serum (Steindl et al. 2021; Haslam et al. 2022). We could expect reports from designated toxicological laboratories with a latency of days (Steindl et al. 2021). Such laboratories can determine the agent from biomedical and environmental samples, but this information is late and has mainly forensic value. According to Mirzayanov, it may be possible to affiliate the agent with a particular laboratory by determining the so-called promotor, i.e., the third component (catalyst or stabilizer) added to binary substances (Lenta 2018).

Iranian scientists were the first to publish analytical data on A-series compounds (Hosseini et al. 2016, 2021; Eskandari et al. 2022). They used the microsynthesis mentioned above to produce deuterated analogs of A-series agents and performed mass spectrometric (MS) analysis via electron ionization and positive electrospray ionization methods. They observed that the ionization process of the studied compounds induced several fragmentation pathways, including McLafferty rearrangement, hydrogen rearrangement, and intramolecular electrophilic aromatic substitution in some cases. Later, they revealed product ion mass spectra of A-230, A-232, A-234, other A-series analogs, and selenophosphorus compounds that they provided to the Central Analytical Database maintained by the OPCW (OCAD) to improve MS detection. The database assists in verification and on-/off-site analysis (Hosseini et al. 2016, 2021; Eskandari et al. 2022). Vibrational spectra modeled by Tan et al. (2019) and nuclear magnetic resonance (NMR) characteristics reported by Jeong et al. (2022b) and Jung et al. (2023) can also be exploited.

The first manuscript utilizing optical detection of chemical warfare agents has already been published. Bauer et al. (2023) used three commercially available handheld forensic light sources to identify contaminated surfaces. They showed that blue light (445 nm peak emission) most effectively visualized auxochromes, including the P(O)N = arrangement in A-series compounds. Although non-specific (not providing a stand-alone identification) and limited to ambient light conditions, a person wearing wavelength filter goggles could rapidly screen surface contamination on-site. Another fast on-site detection method has been developed by Termeau et al. (2023). They presented a rapid, portable, and specific colorimetric detection of A-series compounds based on simple contact with a detection paper. The chemosensor is glass fibers impregnated with hydrazone derivatives. The sharp color contrast is easily observable almost immediately with the naked eye. Simultaneously, the detection apparatus does not respond to other interfering compounds, including other chemical warfare agents.

**Fig. 7:**
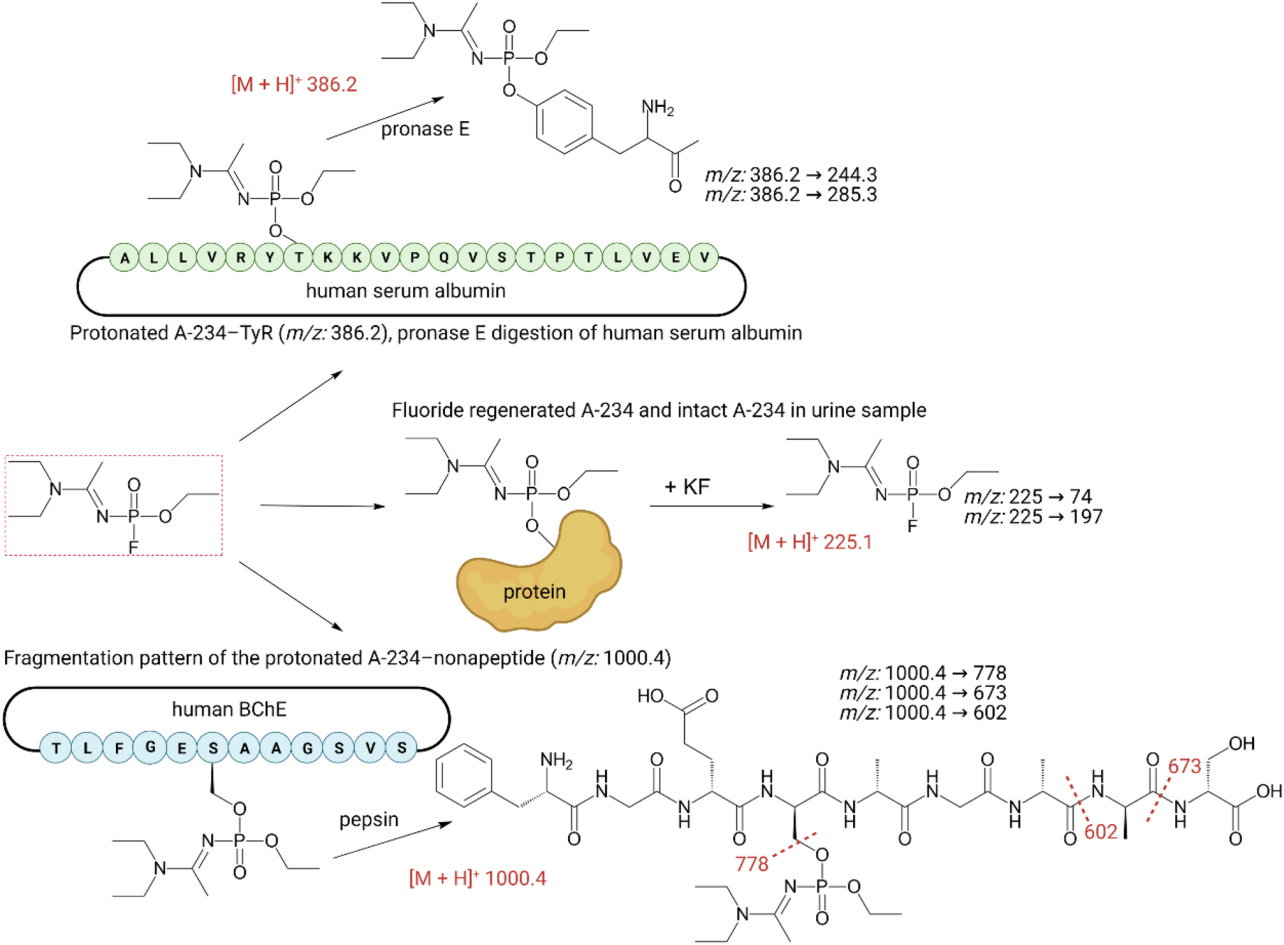
Unambiguous detection and identification of biomarkers of the nerve agent A-234 in biological samples

Another six studies have been published, focusing on identifying A-series compounds in blood and urine (Fig. 7). Jeong et al. (2021) and Noort et al. (2021) applied the nonapeptide method in spiked blood. The technique exploits selective isolation of human BChE from plasma followed by enzymatic cleavage with pepsin, producing the nonapeptide fragment from the active site modified with the stable NA adduct in the case of exposure. Both teams then used precursor ion scanning combined with high-resolution mass spectrometry (HRMS), providing information on the molecular structure of the adduct moiety without reference samples. Jeong et al. (2021) analyzed A-232 and A-234, while Noort et al. (2021) studied A-230, A-232, and A-234. Consistently, they confirmed high protonation of A-series molecules due to the presence of several nitrogen atoms in their structure and unique MS2 fragmentations. Lee et al. (2022) studied the ability of A-234 to react with human serum albumin (HSA) using nano-liquid chromatography (nano-LC)–MS/MS. OP and OP-like compounds can modify HSA at up to 12 sites. However, according to the results, A-234 binds only to Tyr411. Mirbabaei et al. (2022) successfully detected and quantified A-234 in plasma and urine samples by four different GC–MS/MS and LC–MS/MS approaches. They directly measured A-234 released from deactivated proteins using potassium fluoride in plasma and conducted the nonapeptide method and analysis of albumin covalent adducts. In the urine samples, they focused on the targeted detection of the agent in its original form due to the high resistance of A-234 to hydrolysis. The study renders detection limits and calibration curves with other analytical parameters for each method. By contrast, Yamaguchi et al. (2022) and Otsuka et al. (2022) aimed at hydrolytic degradation products of all six A-series compounds reported by Mirzayanov in spiked urine. The degradation products were directly synthesized. Using derivatization, Yamaguchi et al. (2022) validated a novel 4-(4,6-dimethoxy-1,3,5-triazin-2-yl)-4-methylmorpholinium chloride LC–MS/MS method. They detected various phosphoric and phosphonic acids, but the technique was ineffective for the A-242 degradation product. The latter study showed that HILIC–MS/MS analysis is more sensitive to guanidine analogs (A-242 or A-262) (Otsuka et al. 2022). Nevertheless, we must consider the limitations imposed by the fact that the in vivo degradation pathways and pharmacokinetics remain unknown.

Finally, Bruin-Hoegée et al. (2022) and Lee et al. (2023) released an environmental analysis of A-agents. Lee et al. (2023) developed a method based on solid-phase extraction for unknown samples collected from suspect sites. They applied and validated the LC–MS method to analyze the A-234 agent degradation product in water, sand, and soil matrices. Bruin-Hoegée et al. (2022) detected A-234 protein adducts in basil, bay laurel, and stinging nettle leaves using LC–MS/MS. According to their results, biomarkers could be found in the living and dried plants even three months after the exposure, providing a long investigation window.

### Decontamination and therapy

The care of victims affected by A-series agents should not generally differ from that applied to victims of other OP intoxications. The management of patients relies on decontamination, evacuation, proper supportive care, and specific treatment. For all procedures, the stability of A-series agents in the environment (Harvey et al. 2020; Jeong et al. 2021; Jacquet et al. 2021; de Koning et al. 2022; Jung et al. 2023) emphasizes that rescuers, medical caregivers, and other personnel in contact with the victim must wear suitable personal protective equipment to avoid unprotected contact with potentially contaminated surfaces and mitigate any potential cross-contamination. The victim from 2018 (a 41-year-old man; Table 6) was a policeman poisoned by touching a contaminated door handle, wearing only forensic gear (Osborne 2018).

Decontamination is another critical issue. Decontamination based on absorption has not been tested. By contrast, Jung et al. (2023) demonstrated that A-234 was decontaminated by 0.5–1 M mixtures of Oxone^®^ (KHSO_5_·½KHSO_4_·½K_2_SO_4_), Ca(OCl)_2_, KOH, NaOH, and HCl within 30 min. Oxone® and Ca(OCl)_2_ were also successfully tested against A-232. However, such solutions are highly caustic, which could limit their practical use. Additionally, the decontamination efficiency of Oxone^®^ decreased when applied to contaminated sand. Other data on the efficacy of emergency service decontamination mixtures are not publicly available. Only general information and recommendations published by various scientific teams or governments are available. For details, see CHEMM: chemm.nlm.nih.gov; US Army Medical Research Institute of Chemical Defense: ccc.apgea.army.mil; NHS (National Health Service): england.nhs.uk; PHE (Public Health England): publishing.service.gov.uk; and NATO, AMedP-7.1 (Allied joint medical publication): coemed.org or nso.nato.int.

Information on the treatment can be derived only from one publication. Since case reports on incidents from Salisbury and Amesbury are missing, German scientists documented the therapy of the victim poisoned in 2020 (Table 6). Although the therapeutic intervention during the first two days remains unclear, Steindl et al. (2021) assumed that supportive care, particularly intubation with mechanical ventilation, had been most likely the critical element, preventing severe hypoxia and leading to the patient´s favorable outcome. In OP poisonings, death is typically caused by respiratory failure resulting from bronchospasms, bronchorrhea, central respiratory depression, and respiratory muscle weakness/paralysis (Robb and Baker 2023). The importance of intubation and mechanical ventilation is exceptionally high in cases of delayed diagnosis, which may happen in A-series agent intoxications due to misdiagnosing (Morris 2021; Haslam et al. 2022). In particular, if the toxidrome is not fully expressed, miosis, reduced consciousness/unconsciousness, and respiratory depression may be mismatched with an opiate overdose. Escalating doses of naloxone (opiate antidote) can help exclude the diagnosis (Schiller et al. 2023). Supportive therapy of A-series agent poisoning may also include analgosedation, myorelaxation, crystalloids, antipyretics, and antibiotics.

Specific treatment is based on three pillars, including atropine, oxime reactivators, and neuroprotective agents. Atropine or other anticholinergics are administered to control symptoms such as bradycardia, bronchoconstriction, and bronchorrhea (so-called 3Bs). The recommended initial dose of atropine ranges from 2 to 6 mg, depending on the severity of intoxication, and can be doubled in at least 5-min intervals until atropinization happens (adequate blood pressure, heart rate ≥ 80/minute, and clear lungs). Additional dosing is titrated depending on the patient’s clinical response. Specific atropine consumption in A-series agent victims has not been disclosed. Steindl et al. (2021) only mentioned that the patient had received atropine for 12 days. Haslam et al. (2022) pointed out that victims from Salisbury had consumed the hospital-wide supply within 24 h. Atropine can also have diagnostic value. Therapeutic response to high-dose atropine highly suggests OP poisoning (Morris et al. 2019), which was the case of the man from Amesbury. By contrast, the second victim (a 46-year-old man) received small doses of atropine for the eye fundus examination, improving his clinical condition. However, this finding did not prompt the therapy for OP poisoning (Kislinskaya 2001).

Oxime reactivators help restore AChE physiological functions by attacking the OP–AChE complex, releasing the active enzyme. Oxime reactivators may also directly counteract nicotinic- and muscarinic-mediated side effects (Milatović and Jokanović 2009; Soukup et al. 2012; Worek et al. 2020; Gorecki et al. 2022). However, the therapeutic window for oximes after OP exposure can be narrow due to the aging of the OP–AChE conjugate (Worek et al. 2016). The German medical team administered 250 mg of obidoxime bolus (i.v.), followed by a continuous application of 750 mg. After one day, the reactivator administration was discontinued because there was no sign of reactivation or the slightest improvement in neuromuscular function (Steindl et al. 2021). This observation corresponds with preliminary data from Salisbury, suggesting that pralidoxime (i.v.) at 30 mg/kg did not reactivate AChE inhibited by the A-234 (Eddleston and Chowdhury 2021). Nevertheless, British clinicians who treated the victims implied that high doses of pralidoxime helped stabilize cardiac parameters and renal function, possibly through non-targeted interactions with extrasynaptic cholinergic receptors (CHEMM 2019; Hatfill 2019). On the other hand, high oxime doses may impair liver functions (Marrs et al. 2007; Pejchal et al. 2008; Horn et al. 2023). Steindl et al. (2021) observed elevated transaminases and γ-glutamyl transferase several days after they stopped the oxime therapy. However, it is difficult to determine whether this was related to obidoxime, the poisoning, or both.

Neuroprotective agents are necessary to prevent or control seizures as the risk of seizures significantly increases in patients with OP intoxication, and untreated seizures can lead to death (Chuang et al. 2019). Additionally, excessive neuronal activity can induce brain damage and contribute to long-term neurological complications (Pulkrabkova et al. 2023). Benzodiazepines are considered the first-line drugs. Midazolam and diazepam have been approved by the Food and Drug Administration (FDA) for OP poisoning therapy (Jett and Spriggs 2020). Benzodiazepines can also be indicated for OP-induced agitation and delirium (Hui 2018). Interestingly, Steindl et al. (2021) supplemented analgosedation (sufentanil and propofol) with midazolam to support neuroprotection, even though propofol and midazolam have a similar mode of action (Patki and Shelgaonkar 2011).

Finally, fresh frozen plasma, iron, and folate were indicated in the last A-series agent victim (Steindl et al. 2021). Fresh frozen plasma restores BChE levels. BChE can act as a stoichiometric scavenger during the early phase of poisoning (Allard et al. 2022). The patient received six units of fresh frozen plasma on day 6. The administration was prompted by persistently reduced enzyme activity, possibly indicating ongoing redistribution of NA from the lipid tissue into the bloodstream. However, the effectiveness of the infusion, immediately increasing BChE activity, did not confirm this suspicion. On the other hand, the AChE activity was restored much later (after 21 days), suggesting de novo synthesis of the enzyme. From this point of view, the key strategy is to ensure vital functions until the AChE activity is restored; in the case of A-series agents, this means “resynthesized,” as there is no proof that currently available oximes are capable or reactivation of the enzyme. I.v. iron and p.o. folate supplementation helped recover reduced erythrocytes and hemoglobin (Steindl et al. 2021). Other drugs recommended for OP poisoning, including NMDAR and other glutamatergic inhibitors, neurosteroids, magnesium sulfate, lipid emulsion, and antioxidants, may help stabilize the victim or even improve the prognosis (Hoegberg and Gosselin 2017; Vanova et al. 2018; Pulkrabkova et al. 2023). However, preclinical experiments confirming their efficacy are necessary.

## Conclusion

The little information available on the A-series agents indicates that the reviewed compounds represent a unique subgroup of NAs. The situation is further complicated by the emergence of other alternative names, such as fourth-generation agents (FGAs) (Konopski 2009; Halamek and Kobliha 2011) or non-traditional agents (NTAs) (Meselson and Robinson 2005). Unification of nomenclature will, therefore, play an important role. Another problem is the public data availability on their properties, structures, and toxicities. Such information is minimal, primarily based on computational studies or classified. These agents present several unique challenges regarding toxicity, such as detection, persistence, decontamination, treatment, and the potential for delayed onset of symptoms. There is no proof that marketed oximes can reactivate inhibited AChE. Therefore, only symptomatic treatment, consisting of parasympatholytic and neuroprotective agents, is in hand. If such poisoning is recognized, supplementation by BChE-containing plasma may lead to scavenging of the poison. In any other case, ensuring the vital function is crucial until the replenishment of the AChE pool. Nevertheless, more research will be necessary to overcome these challenges. However, given the hazardous nature and legislative constraints, current research is conducted in a limited number of laboratories. It is also uncertain to what extent the results will be shared. Therefore, new data will most probably emerge very slowly.
